# Analysis of Chronic Mild Stress-Induced Hypothalamic Proteome: Identification of Protein Dysregulations Associated With Vulnerability and Resiliency to Depression or Anxiety

**DOI:** 10.3389/fnmol.2021.633398

**Published:** 2021-03-02

**Authors:** Weibo Gong, Wei Liao, Chui Fang, Yanchen Liu, Hong Xie, Faping Yi, Rongzhong Huang, Lixiang Wang, Jian Zhou

**Affiliations:** ^1^Institute of Neuroscience, Chongqing Medical University, Chongqing, China; ^2^Basic Medical College, Chongqing Medical University, Chongqing, China; ^3^Shenzhen Wininnovate Bio-Tech Co., Ltd., Shenzhen, China; ^4^Department of Pharmacy, Chongqing Renji Hospital, University of Chinese Academy of Sciences, Chongqing, China; ^5^ChuangXu Institute of Life Science, Chongqing, China

**Keywords:** anxiety, chronic mild stress, depression, hypothalamus, quantitative proteomics

## Abstract

Chronic stress as a known risk factor leads to hyperactivity of the hypothalamus-pituitary-adrenal (HPA) axis in both depression and anxiety. However, the stress-induced dysfunction of the HPA axis in these disorders especially the common and unique molecular dysregulations have not been well-explored. Previously, we utilized a chronic mild stress (CMS) paradigm to segregate and gain depression-susceptible, anxiety-susceptible, and insusceptible groups. In this study, we continue to examine the possible protein expression alterations of the hypothalamus as the center of the HPA axis in these three groups by using a proteomic approach. Though isobaric tags for relative and absolute quantitation (iTRAQ)-based quantitative analysis, a total of 593 dysregulated proteins were identified. These were potentially associated with vulnerability and adaptability of CMS-caused depression or anxiety and therefore might become novel investigative protein targets. Further independent analysis using parallel reaction monitoring (PRM) indicated that 5, 7, and 21 dysregulated proteins were specifically associated with depression-susceptible, anxiety-susceptible, and insusceptible groups, respectively, suggesting that the same CMS differently affected the regulation system of the rat hypothalamic proteome. In summary, the current proteomic research on the hypothalamus provided insights into the specific and common molecular basis for the HPA dysfunction mechanisms that underlie resiliency and vulnerability to stress-induced depression or anxiety.

## Introduction

Depression and anxiety are two common and chronic mental illnesses that negatively affect the social interactions, career, and well-being of patients, families, and society (Larson et al., 2007; Almeida et al., 2012; Hamilton et al., 2015). Clinically, depression and anxiety disorders show different core symptoms, but they usually coexist (Brodbeck et al., 2011; Melton et al., 2016; Yun et al., 2016). Due to their considerable overlap in comorbidities and pathophysiology, most clinical and basic investigation data were frequently mixed, which may obscure the understanding of the factors that regulate these two disorders (Chiba et al., 2012; Yun et al., 2016). Consequently, some recent studies are beginning to separately investigate non-complicated individuals with the two diseases to unravel the common and distinct features of the central nervous systems (Lotan et al., 2014; Frick, 2017; Zhao et al., 2017; Chen et al., 2018).

Anxiety and depression are two complex and heterogeneous disorders. Numerous studies have shown that there are common risk factors between these two disorders, such as life event stress and chronic stress (Pittenger and Duman, 2008; Mathew et al., 2011; Leuner and Shors, 2013). Increasing evidence shows that chronic stressful life events such as deleterious environmental factors cause anxiety and depression (Chang and Grace, 2014; Yun et al., 2016). However, even when exposed to chronic stress, many individuals do not show symptoms of anxiety and depression (Krishnan et al., 2007; Uchida et al., 2011; Henningsen et al., 2012; Russo et al., 2012). To model some environmental factors that affect humans, researchers commonly subject rodents to chronic mild stress (CMS) in order to generate behaviors that are useful for the study of depression- and anxiety-like disorders (Henningsen et al., 2012; Chang and Grace, 2014). To illustrate the potential biological cause and pathophysiology of the two disorders, the focus on the neural and molecular substrates that are sensitive and adaptable to the stress-induced diseases will be very significant (Uchida et al., 2011; Henningsen et al., 2012; Russo et al., 2012; Chang and Grace, 2014).

Mounting studies have illustrated that depression and anxiety as stress-related disorders involve hyperactivity of the hypothalamic-pituitary-adrenal (HPA) axis (Lucassen et al., 2013; Renoir et al., 2013; Borrow et al., 2016). As the key brain region of the HPA axis, the hypothalamus plays an important role in the physiological stress response (Pittenger and Duman, 2008; Rao et al., 2016). Hypothalamic and limbic areas coordinate cognitive, emotional, neuroendocrine, and autonomic inputs in the classic neuroendocrine circuit, and jointly determine the specificity and magnitude of the behavioral, neural, and hormonal responses of an individual to stress (Lucassen et al., 2013). Brain imaging studies of depressive and anxious disorders have indicated that the volume of the hypothalamus as well as its interactions and coherence were altered by stress (Dedovic et al., 2009; Pruessner et al., 2010; Sousa and Almeida, 2012; Oliveira et al., 2013). This stress-induced hypothalamic morphological change inevitably causes cellular and molecular dysfunctions in depression and anxiety (Sousa and Almeida, 2012). Although the hypothalamic plasticity is potentially abnormal in the two disorders, the corresponding molecular basis may be intrinsically different and still obscure. Therefore, the identification of common and unique molecular characteristics of mal-adaptability and adaptability to depression or anxiety has become an urgent need.

In our previous study, we quantitatively profiled the hippocampal proteome to discover common and unique protein components correlated to anxiety-like and depression-like behavioral phenotypes induced by CMS (Tang et al., 2019). Through the behavioral tests, the depression-susceptible, anxiety-susceptible, and insusceptible groups were segregated as the three different responses to stress (Tang et al., 2019). To probe continuously into stress-related anxiety and depression, in the current study we utilized the hypothalamic tissues from the same batch of our previously-established CMS rat model. The proteomes of the hypothalamus from the three stressed groups and the control group were comparatively analyzed, offering the significant molecular basis associated with maladaptive and adaptive behavioral phenotypes to anxiety or depression. Our proteomic data could provide a window into the understanding of the common and distinct molecular mechanisms underlying stress resistance and stress-induced anxiety or depression.

## Materials and Methods

### CMS Rat Model and Behavioral Testing

The animal experiments in our study were approved by the Ethics Committee of Chongqing Medical University. All procedures were performed in accordance with the National Institutes of Health protocols for the use and care of laboratory animals. We purchased male Sprague-Dawley albino rats with a bodyweight of approximately 250 g from the local animal center. We individually housed all rats and allowed them *ad libitum* access to food and water. The rats were maintained under the following conditions: the relative humidity was 55 ± 5%; the room temperature was 21–22°C with a 12 h light/dark cycle.

As described previously (Tang et al., 2019), we conducted the CMS procedure after the rats were habituated to sucrose consumption. Following a baseline sucrose preference test, the rats were randomly allocated to the control group and stressed group. Rats in the control group were provided with standard daily care. Those in the stressed group were exposed to the 8 week CMS procedure, which involved a variety of stressors; namely, strobe light, continuous lighting, white noise, paired housing, a 45° cage tilt, a soiled cage, water deprivation, and an empty water bottle.

During this CMS exposure, depression-like behavior (anhedonia) of the rats was assessed using the sucrose preference test (SPT) as previously described (Tang et al., 2019). We utilized the formula to calculate sucrose preference: sucrose preference = (sucrose intake/total fluid intake) × 100%. Then, we performed a forced swimming test (FST) to evaluate the despair-like behavior of the rats using a water-filled Plexiglas cylinder and a video monitoring system as described previously (Tang et al., 2019). At the same time, we employed an elevated plus maze test (EMT) to determine the rat anxiety-like behavior as illustrated previously (Tang et al., 2019). Also, we recorded the animal body weights every week during this process.

### Extraction and Digestion of Hypothalamic Tissue Proteins

After the behavioral tests were completed, the rats were sacrificed and their whole brains were excised rapidly. The hypothalamus tissues were dissected from the brain, and quickly frozen in liquid nitrogen, and kept at −80°C. To extract hypothalamic proteins, we homogenized the tissues with SDT lysis buffer (100 mM Tris–HCl, pH 8.0, 100 mM dithiothreitol, 4% SDS, and protease inhibitors). The resulting extracts were heated at 100°C for 5 min. After centrifugation, the protein contents in the supernatant were measured with the Pierce bicinchoninic acid assay method.

Afterward, we used filter-aided sample preparation (FASP)-based method to digest the proteins. As in our previously-described procedure (Qiao et al., 2017), the 10 kD ultrafiltration centrifuge tubes were utilized for effective protein digestion. In brief, the protein solutions were diluted by adding urea buffer containing 8 M urea, 150 mM Tris-HCl, pH 8.0, and subsequently alkylated in the dark using 50 mM iodoacetamide for half an hour. The protein solutions were centrifuged and washed twice by adding the urea buffer. Then the proteins were incubated overnight with trypsin at 37°C. After centrifugation, the tryptic peptides were obtained by repeatedly washing. The collected peptides were lyophilized in a Speed Vac.

### Peptide iTRAQ Labeling and High-pH Reversed-Phase Liquid Chromatography (RPLC) Fractionation

We labeled the tryptic peptides using 8-plex iTRAQ reagents as described previously (Tang et al., 2019). Eight samples from the three stressed groups (depression-susceptible, anxiety-susceptible, and insusceptible) and the control group were labeled with the iTRAQ reagents 113–121. Each sample was from two or three rats in each group. After the peptides were labeled, the eight samples were pooled and then fractionated utilizing off-line high-pH RPLC. The peptide mixture was separated by 80 min gradient elution, in which the high-pH buffer (25% ACN, 10 mM ammonium formate, 90% acetonitrile, pH 10.0) was linearly increased from 5 to 38% at a flow rate of 0.3 ml/min. The collected sixteen fractions were desalted and dried for the following liquid chromatography-tandem mass spectrometry (LC-MS/MS) analysis.

### Q-Exactive LC-MS/MS Analysis

The labeled peptides were re-dissolved with 0.1% formic acid and then loaded into a nanoViper trapping column (C18, 3 μm, 100 Å). A Thermo Scientific Easy-nLC 1200 system was employed for the online chromatographic separation of peptides. The peptides were effectively desalted by elution of 100% solution A (0.1% formic acid). Subsequently, we used an analytical column (50 μm × 150 mm, 3 μm C18 100 Å) to separate the peptides along with a 50 min elution gradient. In the gradient, solution B (0.1% formic acid/80% acetonitrile) was linearly increased from 8 to 38%. Afterward, the MS analysis was conducted on a Q-Exactive Orbitrap mass spectrometer (Thermo Fisher Scientific) equipped with a Nano Flex ion source. For the MS operation, a 275°C interface heater temperature and a 1.9 kV ion spray voltage were used. The tandem MS data were acquired by using a data-dependent acquisition mode with full MS scans, where MS1 spectra were collected in the m/z range from 350 to 1,200, and MS2 spectra were collected in the m/z range from 110 to 1,200. The 250 ms survey scans were acquired along with up to fourteen MS/MS product ion scans of 50 ms. MS spectra with two and above charge-state were selected and fragmented by using higher-energy collision dissociation. We set the dynamic exclusion time for 25 s.

### Data Analysis

We employed the software Proteome Discoverer (version 2.1, ThermoFisher) with the Sequest HT search engine for identification and quantitation of peptides and proteins. The UniProt_Rat release 2017_12 database was introduced into the software. The search parameters were as follows: two trypsin missed cleavages, iTRAQ 8plex on N-term and Lys and Carbamidomethylation on Cys as fixed modifications, Oxidation on Met, acetylation on protein N-term, deamidation on Asn and Gln, and Pyro-Glu as variable modifications, precursor fragment mass tolerance of 10 ppm, and fragment mass tolerance of 0.05 Da. For peptide and protein identifications, we used the Percolator module to control the peptide-spectrum-match false discovery rate (FDR) below 1.0%, as described previously (Han et al., 2015). Further, we utilized Reporter Ions Quantifier and Peptide and Protein Quantifier nodes to calculate the relative ratios of peptides and proteins across samples. The quantified data were introduced to Microsoft Excel for manual processing. Then, the protein ratios were analyzed by a two-tailed Student’s *t*-test. Those proteins with a 1.2-fold alteration and *p*-values less than 0.05 were deemed to be significant differences, as used in previous studies (Tian et al., 2018; Zhang et al., 2021). The raw data have been deposited to the ProteomeXchange Consortium^1^ via the iProX partner repository (Ma et al., 2019) with the dataset identifier PXD022715.

### Bioinformatics Analysis

The differential protein data were analyzed with the OmicsBean tool^2^ to obtain the Gene Ontology (GO)^3^ terms, as described previously (Xie et al., 2018; Tang et al., 2019). The GO terms in biological processes (GOBP), molecular functions (GOMF), and cellular components (GOCC) were enriched. A Kyoto Encyclopedia of Genes and Genomes (KEGG)^4^ pathway analysis was performed using the same tool, in which a *p*-value of less than 0.05 was deemed significant. Furthermore, we evaluated protein-protein interaction (PPI) by use of the database of the Search Tool for the Retrieval of Interacting Genes/Proteins (STRING). The acquired network was further visualized using Cytoscape software, as described previously (Tang et al., 2019).

### Parallel Reaction Monitoring (PRM) Analysis

Following the iTRAQ experiment, the sample preparation including protein extraction and digestion were carried out. The obtained peptides were injected into the Q-Exactive mass spectrometer and analyzed through LC-MS/MS. The 28 normalized collision energy was used for peptide fragmentation. The resulting fragments were examined in the Orbitrap at a resolution of 35,000. The raw data were acquired and subsequently analyzed by using the software Proteome Discoverer. Skyline software (version 19.1) was employed to process the MS data.

### Statistical Analysis

SPSS software was used for the data statistical analysis. All data were subjected to Student’s *t*-test and expressed as means ± standard error. The results with *p*-values less than 0.05 were deemed statistically significant.

## Results

### Hypothalamic Proteomics Analysis of the CMS Model Rats

**FIGURE 1:**
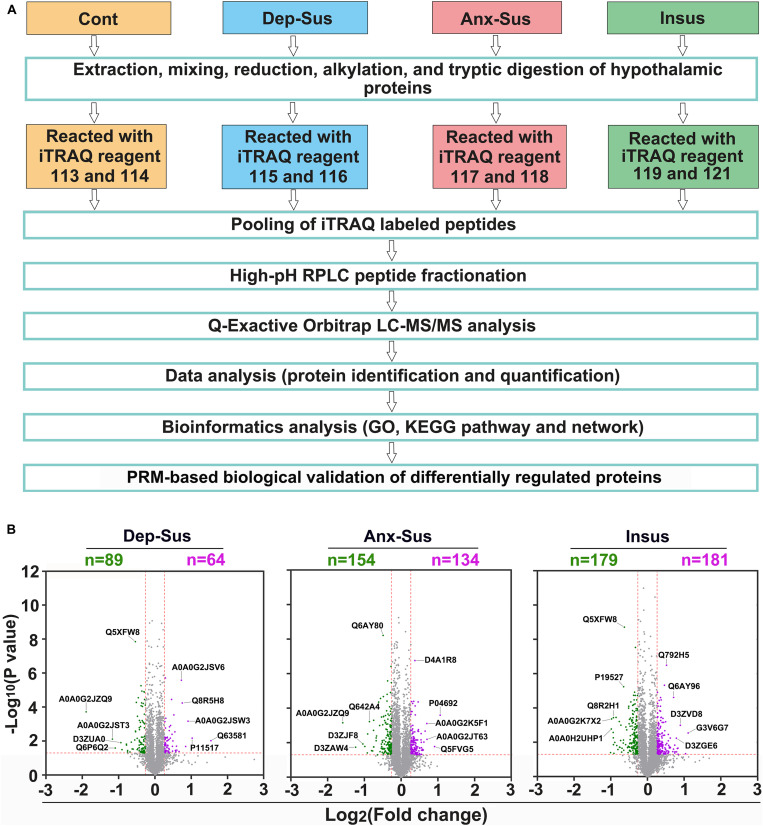
Hypothalamic proteome profiling of the control (Cont), depression-susceptible (Dep-Sus), anxiety-susceptible (Anx-Sus), and insusceptible (Insus) groups. **(A)** Flow chart for the quantitative proteomic analysis. **(B)** Analysis of differentially regulated proteins in the hypothalamus. Volcano plot showing protein expression alternations in the Dep-Sus, Anx-Sus, and Insus groups. The X-axis represents the log2 fold-change, and the y-axis represents the log10 negative *p-*value. Green and purple, respectively, illustrate the low and high relative expressions. Examples of proteins that are differentially expressed with high significance are indicated with their Uniprot accessions.

In the present study, SPT and FST were first used to evaluate the depression-like behavior (i.e., anhedonia and behavioral despair) induced by CMS. Then we conducted the EMT experiment to index the anxiety-like behavior. Taking into account the hypothalamic sample used from the same batch of CMS-induced rat model in our previous publication (Tang et al., 2019), thereby it was briefly mentioned that, compared with the other groups, the sucrose preference of the depression-susceptible group was reduced, while the immobility time was increased. As expected, some stressed rats did not exhibit depression-like behavior. Interestingly, we observed their anxiety-like behavior based on the EMT data and consequently defined them as an anxiety-susceptible group. Additionally, the SPT, FST, and EMT data of the insusceptible group were not significantly changed relative to the control group. This indicated that these insusceptible rats did not have any depressive and anxious behaviors. It should be mentioned that the body weight change of the insusceptible group was significantly different from that of the control group during 8 weeks, but similar to those of the other two stressed groups (Tang et al., 2019). Through these assessments, a subset of the control, depression-susceptible, anxiety-susceptible, and insusceptible groups were gained. These behavioral data indicated that the CMS procedure provided an effective preclinical model for exploring the molecular patterns associated with the vulnerability and adaptability of depression or anxiety induced by CMS. Next, we conducted the quantitative proteomics analysis on the hypothalamic tissues of the four groups. As illustrated in Figure 1A, the hypothalamic proteomes of the depression-susceptible, anxiety-susceptible, insusceptible, and the control groups were comparatively profiled by using iTRAQ-based quantitative strategy. The proteomics experiment was performed on five rats per group. As reported previously (Lenselink et al., 2015), we pooled with an equal amount of the hypothalamic proteins randomly taken from two or three individuals for each sample. As a result, a total of 4,181 non-redundant proteins were identified and quantified along with the FDR less than 1% (Supplementary Table S1). By comparing the hypothalamic proteomes of the four different groups, we identified a total of 593 CMS-responsive proteins using a cut-off of 1.2-fold change and a *p-*value of less than 0.05 (Figure 1B). As described in previous studies (Baughman et al., 2001; Andrews et al., 2004; Scagliotti et al., 2008; Bonati et al., 2015), we treated all proteomics data as independent hypotheses, and did not adjust for multiple comparisons. The results having *p-*values less than 0.05 can be taken to be suggestive of trends in hypothalamic protein expression changes (Zhang et al., 2021). A *p-*value smaller than this would correspond to a stronger suggestion. Herein, the proteomics profile of the hypothalamus was compared with that of the hippocampus from our previous study (Tang et al., 2019; Supplementary Figure S1). Although the overall proteins quantified in both the hippocampus and hypothalamus showed a similar profile with a 42–68% overlap, the sets of differentially regulated proteins of each region were considerably divergent, suggesting that the two regions had the different proteomic responses to the CMS.

### Functional and Network Characterization of CMS-Affected Dysregulated Proteins

**FIGURE 2:**
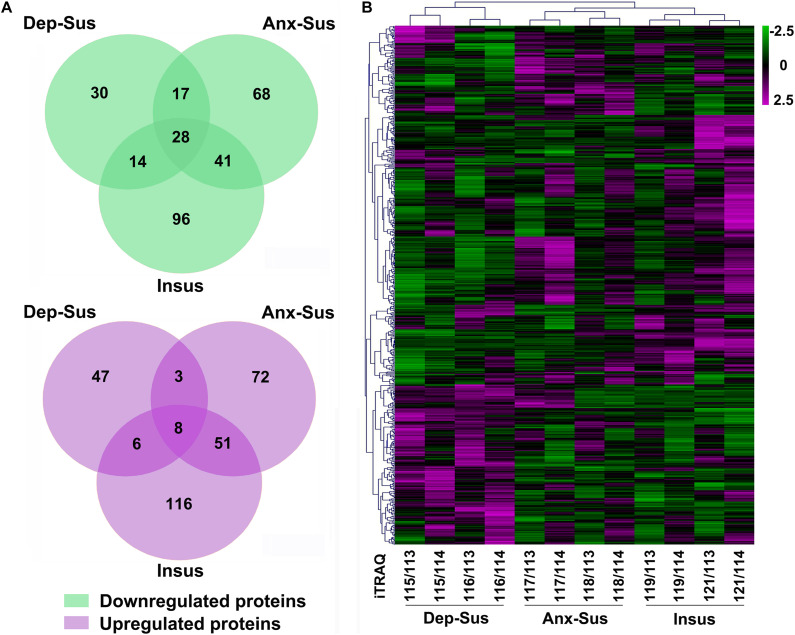
Analysis of the differentially regulated proteins from the depression-susceptible (Dep-Sus), anxiety-susceptible (Anx-Sus), and insusceptible (Insus) groups. **(A)** Venn diagrams displaying the number of down-regulated and up-regulated proteins. Detailed information can be seen in Supplementary Table S2. **(B)** Heatmap-based clustering of differentially regulated proteins identified in the three groups. Lower expressions are indicated with green and higher expressions with purple. The intensities of various colors illustrate the expression levels. The color bar is log2 scaled.

Through rat hypothalamic proteomics analysis, we determined that 593 proteins were differentially regulated under the CMS. Among these proteins, 89 proteins were down-regulated and 64 proteins were up-regulated in the depression-susceptible group, 154 down-regulated and 134 up-regulated in the anxiety-susceptible group, and 179 down-regulated and 181 up-regulated in the insusceptible group (Figure 2A and Supplementary Table S2). We observed 56 proteins with similar regulations in the two stress-susceptible groups, which may represent some common abnormal components between depression and anxiety. Furthermore, among the two susceptible and the insusceptible groups, 148 proteins were found to be similarly regulated as a result of CMS exposure. Interestingly, up to 72% of these dysregulated proteins were specifically related to these three behavioral phenotypes, suggesting that the three stress-related groups had different molecular features under the CMS. Notably, the differential proteins uniquely from the insusceptible group represented several potential molecular adaptations within the hypothalamus (Krishnan et al., 2007; Henningsen et al., 2012). Further unsupervised hierarchical cluster analysis of 593 dysregulated proteins divided them into three different groups, which also reflected the three different responses to CMS (Figure 2B).

**FIGURE 3:**
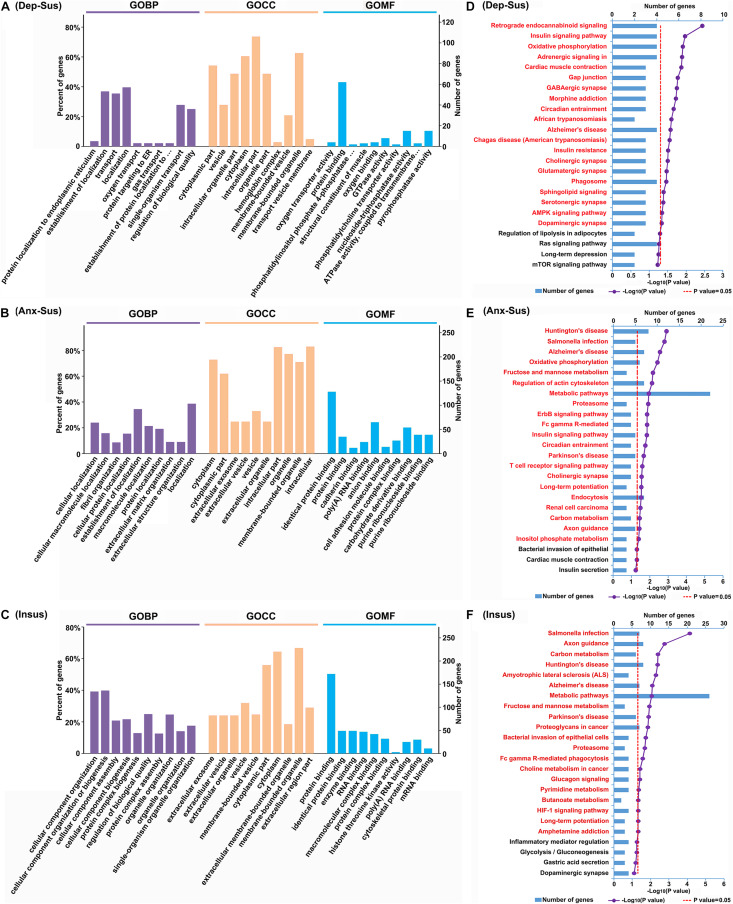
Gene Ontology (GO) and Kyoto Encyclopedia of Genes and Genomes (KEGG) pathway enrichment analysis of the differentially regulated proteins from the depression-susceptible (Dep-Sus), anxiety-susceptible (Anx-Sus), and insusceptible (Insus) groups. **(A–C)** The top 10 enriched GO biological process (GOBP), cellular component (GOCC), and molecular function (GOMF) terms are indicated. **(D–F)** The significantly enriched KEGG pathway terms are showed by titles in red. The X-axis represents the log10 negative *p*-value.

To gain an in-depth understanding of significant biological functions and pathways related to behavioral phenotypes, we used the OmicsBean tool to investigate these dysregulated proteins in the three groups. Based on the GO and KEGG pathway databases, the 153 differentially regulated proteins in the depression-susceptible group were analyzed (Supplementary Table S3). As a result, a total of 447, 158, 137, and 20 terms in the GOBP, GOCC, GOMF, and KEGG pathway categories were identified to be significantly overrepresented, respectively. Herein we showed the top 10 enriched GO terms (Figure 3A). The GOBP analysis indicated that many proteins were involved in protein localization and targeting to the endoplasmic reticulum, localization, and transport. Most proteins in the GOCC category belonged to vesicle, cytoplasmic, intracellular, and organelle parts. GOMF category analysis showed that the majority of proteins were involved in transporter and enzyme activity, and protein and oxygen binding. The enrichment analysis of KEGG pathway showed that these dysregulated proteins were mainly involved in retrograde endocannabinoid, adrenergic, sphingolipid, and AMPK signaling, and GABAergic, cholinergic, glutamatergic, serotonergic, and dopaminergic synapse pathways (Figure 3D).

At the same time, we conducted the GO and KEGG pathway enrichment of 288 proteins differentially regulated in the anxiety-susceptible group. A total of 829 GOBP, 223 GOCC, 194 GOMF, and 21 KEGG pathway terms were identified to be significantly enriched. The top 10 enriched GO terms were indicated (Figure 3B). The analysis of GOBP classification displayed that most proteins were related to cellular, macromolecule, and protein localization, and fibril, extracellular matrix, and structural organization. The analysis of GOCC category showed that most of the dysregulated proteins belonged to cytoplasmic, intracellular and organelle parts, extracellular exosome, organelle, and vesicle, while GOMF indicated most proteins were related to protein, RNA, anion, cell adhesion molecule, protein complex, carbohydrate derivative, purine ribonucleoside, and nucleoside binding. The analysis of KEGG pathways revealed these differential proteins were mainly associated with Huntington’s, Alzheimer’s, and Parkinson’s diseases, metabolic, signaling, and synapse pathways (Figure 3E).

After that, we also enriched GO and KEGG pathway terms of 360 differentially regulated proteins from the insusceptible group. As a result, a total of 997 GOBP, 228 GOCC, 191 GOMF, and 20 KEGG pathway terms were identified to be significantly overrepresented. The top 10 overrepresented GO terms were shown in Figure 3C. GOBP category analysis showed that most proteins were involved in cellular component organization, biogenesis and assembly, protein complex biogenesis, assembly and subunit organization, and organelle organization. The GOCC classification analysis indicated that the majority of proteins were located in the extracellular exosome and organelle, vesicle, and cytoplasm. It was predicted that most proteins in the GOMF category were involved in protein, RNA, macromolecular and protein complex binding, and enzyme activity, and the enrichment of KEGG pathways demonstrated that these dysregulated proteins were mainly related to metabolic, signaling, Huntington’s, Alzheimer’s, and Parkinson’s disease pathways (Figure 3F).

In addition, GO and KEGG pathway terms of 36 proteins that were commonly affected in all three groups were enriched. A total of 221 GOBP, 55 GOCC, 49 GOMF, and 9 KEGG pathway terms were identified to be significantly enriched (Supplementary Table S4). From the top 10 enriched GO terms, we observed that the majority of proteins in the GOBP category were classified into catabolic, biosynthetic and metabolic processes, and regulations of localization and transport; most of these proteins in the GOCC classification belonged to fiber, vesicle, and extracellular exosome and organelle; and most proteins in the GOMF analysis were involved in enzyme activity. KEGG analysis revealed that these proteins were mainly associated with metabolic and signaling pathways. Meanwhile, we also enriched GO and KEGG pathway terms of 56 proteins affected in both of the susceptible groups. A total of 290 GOBP, 103 GOCC, 64 GOMF, and 18 KEGG pathway terms were significantly enriched (Supplementary Table S5). From the top 10 enriched GO terms, it could be observed that most of these proteins in the GOBP analysis were involved in metabolic, catabolic, biosynthetic, and muscle system processes, and establishment of localization; the majority of proteins in the GOCC category were located in the fiber, vesicle, myosin, and actin filament and cytoskeleton; and most proteins in the GOMF classification were involved in catalytic and enzyme activities. The enrichment of KEGG pathways indicated that these proteins were mainly involved in oxidative phosphorylation, metabolic, signaling and synapse pathways.

**FIGURE 4:**
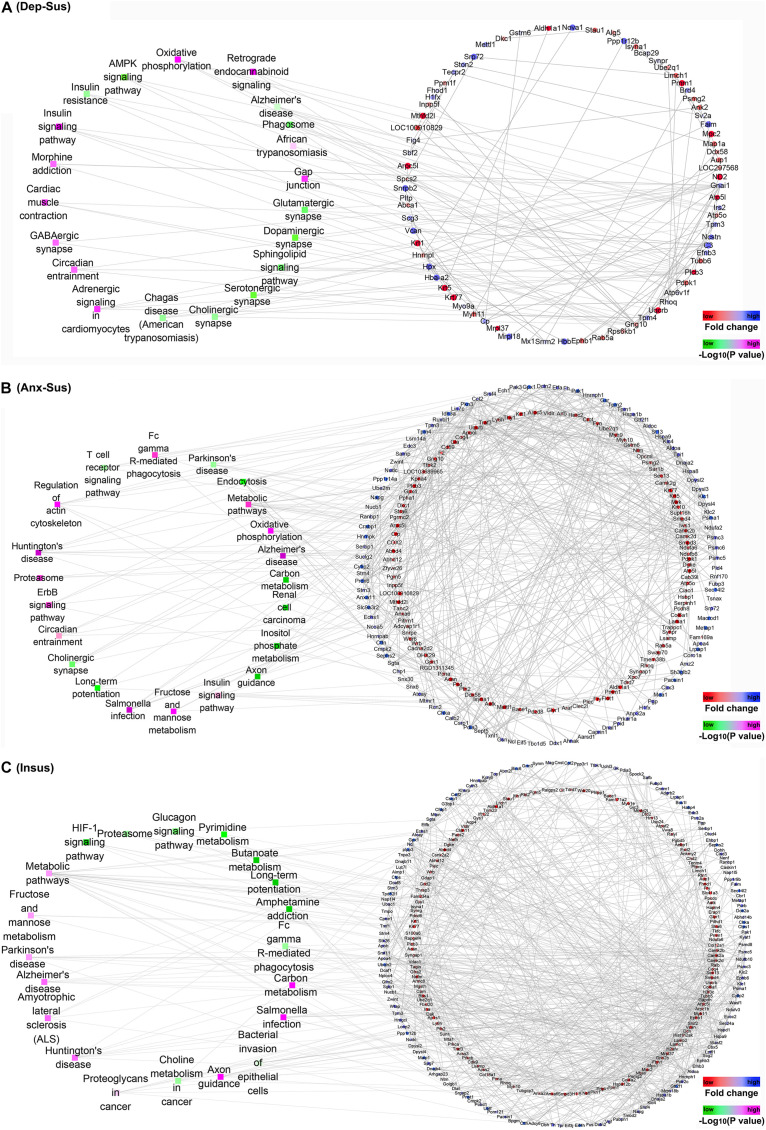
Protein-protein interaction (PPI) analysis of the differentially regulated proteins in the depression-susceptible (Dep-Sus), anxiety-susceptible (Anx-Sus), and insusceptible (Insus) groups. **(A–C)** The PPI networks of the Dep-Sus **(A)**, Anx-Sus **(B)**, and Insus **(C)** groups are built on basis of altered protein expressions and overrepresented Kyoto Encyclopedia of Genes and Genomes (KEGG) pathways. Proteins/genes are indicated with circular nodes, and the KEGG pathways are indicated with the rectangle that is colored according to the *p*-values.

Further, we focused on the protein-protein interaction (PPI) networks inferred by proteomics in the three groups (Figures 4A–C). The networks of the three stressed groups were built by using these dysregulated proteins along with the significantly-enriched KEGG pathways. It could be observed that the PPI network complexity of the anxiety-susceptible and insusceptible groups was higher than that of the depression-susceptible group, which suggested protein interactomes of the former two groups were more heavily affected by the CMS. This might represent differences in important protein dysregulation systems and active biological processes that occurred in these stressed groups. Based on a unified conceptual framework, we identified 16, 39, and 51 proteins as significant nodes in the networks from the depression-susceptible, anxiety-susceptible, and insusceptible groups, respectively. The PPI networks revealed the significant KEGG pathways and the corresponding dysregulated proteins and their close correlations, and thus provided a small pool of interactome that related to the three behavioral phenotypes.

### PRM Analysis of CMS-Related Proteins

**FIGURE 5:**
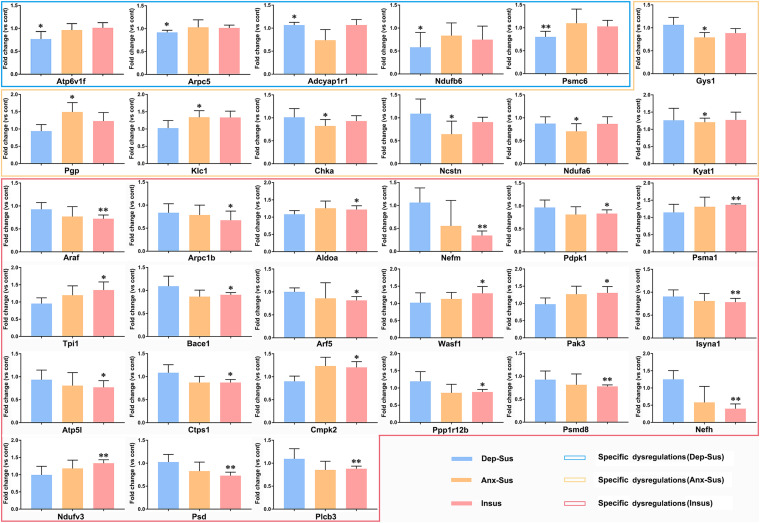
Parallel reaction monitoring (PRM) analysis of the differentially regulated proteins in the depression-susceptible (Dep-Sus), anxiety-susceptible (Anx-Sus), and insusceptible (Insus) groups as compared to the control (Cont) group. The expression levels of Atp6v1f, Arpc5, Adcyap1r1, Ndufb6, Psmc6, Gys1, Pgp, Klc1, Chka, Ncstn, Ndufa6, Kyat1, Araf, Arpc1b, Aldoa, Nefm, Pdpk1, Psma1, Tpi1, Bace1, Arf5, Wasf1, Pak3, Isyna1, Atp5l, Ctps1, Cmpk2, Ppp1r12b, Psmd8, Nefh, Ndufv3, Psd, and Plcb3 were examined on the rat hypothalamic protein extracts and found to be specific dysregulations in the Dep-Sus, Anx-Sus and Insus groups. *n* = 5, *p < 0.05, **p < 0.01.

As an alternative method of targeted quantification, the PRM has been widely applied to validation of multiple proteins in complex sample (Yan et al., 2018; Wu et al., 2019; Zhang et al., 2021). In the current study, we further used the PRM-based approach to independently validate 54 dysregulated proteins of interest from the significantly enriched KEGG pathways and networks. On the whole, it can be observed that the results from the PRM mirrored the iTRAQ data (Supplementary Figure S2). As reported in other proteomics studies (Abdi et al., 2006; Cheng et al., 2011; Xu et al., 2012; Wu et al., 2019), some discrepancies objectively exist between the two iTRAQ- and PRM-based quantitative results. Besides the examination difference of these two approaches (Wu et al., 2019), another reason may be the additional pooling procedure in the iTRAQ proteomics experiment. As shown in Figure 5, the expressions of Atp6v1f, Arpc5, Ndufb6, and Psmc6 were significantly down-regulated while Adcyap1r1 was up-regulated in the depression-susceptible group compared with the control group. It was observed that the expressions of Gys1, Chka, Ncstn, and Ndufa6 were significantly down-regulated whereas Pgp, Klc1, and Kyat1 were up-regulated in the anxiety-susceptible group as compared to the control group. Moreover, we observed that the expressions of Arpc1b, Nefm, Pdpk1, Araf, Bace1, Arf5, Isyna1, Atp5l, Ctps1, Ppp1r12b, Psmd8, Nefh, Psd, and Plcb3 were significantly down-regulated while Aldoa, Psma1, Tpi1, Wasf1, Pak3, Cmpk2, and Ndufv3 were up-regulated in the insusceptible group when compared to the control group. In addition, a reduced level of Uqcrb in both the depression-susceptible and anxiety-susceptible groups, and elevated levels of Echs1 and Hspa8 in both the anxiety-susceptible and insusceptible groups were found as compared to the control group (Supplementary Figure S3). We also observed that the expression levels of Rab5a, Ephb1, Camk2a, Camk2d, Nefl, Tkfc, Fyn, Flot1, and Vdac1 were significantly down-regulated while Pak1, Cbr1, Dpysl2, Klc4, Klc2, Snx6, and Cttn were up-regulated in both the anxiety-susceptible and insusceptible groups compared with the control group.

## Discussion

As an unfavorable factor (Daviu et al., 2019), the CMS was usually employed to produce depressed and anxious behaviors in rodents (Henningsen et al., 2012; Chang and Grace, 2014). In our previous study (Tang et al., 2019), we used a well-established CMS rat model to identify the three different phenotypes (i.e., depression-susceptible, anxiety-susceptible, and insusceptible) based on the behavioral tests. The model supplied a valuable method for determining common and unique molecular characteristics of vulnerability and adaptability to depression or anxiety, which thus contributed to the biomedical translational research.

To unravel the possible protein dysregulations related to the three behavioral phenotypes, we carried out a comparative analysis of the hypothalamic proteomes of the stressed rats by the iTRAQ-based quantitative method. As a result, a total of 593 CMS-affected proteins were identified that were differentially regulated in the three stressed groups as compared to the control group. The overlapping proteome alterations between the two susceptible groups might represent a common molecular pattern of depression and anxiety.Intriguingly, the distinct protein dysfunctional profiles in the three stressed groups might represent differences of the three behavioral phenotypes. This proteome result will help researchers to discover protein systems and pathways related to vulnerability and adaptability to stress-induced anxiety or depression.

Our subsequent bioinformatics analysis suggested affected pathways in the hypothalamus particularly related to the three behavioral phenotypes. The pathway analysis indicated that the differentially regulated proteins were significantly overrepresented for signaling and synapse dysregulations in the depression-susceptible group, metabolism, signaling, synapse, and neuropsychiatric disease-related dysfunctions in the anxiety-susceptible group, and metabolism, signaling, and neuropsychiatric disease-related repercussions in the insusceptible group. Meanwhile, the network analysis revealed the protein dysregulation systems along with these significant pathways, potentially providing some useful clues for the pathophysiological molecular basis of the three phenotypes.

Further, we independently analyzed the 54 differentially regulated proteins involved in the significant KEGG pathways by using the PRM method. The results indicated that Atp6v1f, Arpc5, Adcyap1r1, Ndufb6, and Psmc6 were distinctly dysregulated in the hypothalamus of the depression-susceptible group, while Gys1, Pgp, Klc1, Chka, Ncstn, Ndufa6, and Kyat1 were distinctly dysregulated in the anxiety-susceptible group. The specificity of molecular responses indicated that the same CMS differently affected the molecular functions, correspondingly providing an impetus to diverse biological processes and rat behavioral phenotypes. Intriguingly, the expressions of Arpc1b, Aldoa, Nefm, Pdpk1, Araf, Psma1, Tpi1, Bace1, Arf5, Wasf1, Pak3, Isyna1, Atp5l, Ctps1, Cmpk2, Ppp1r12b, Psmd8, Nefh, Ndufv3, Psd, and Plcb3 were found to be uniquely dysregulated in the insusceptible group, which suggested a potentially positive way to deal with the CMS-affected hypothalamic protein dysfunctions in the rats for stress protection (Krishnan et al., 2007; Uchida et al., 2011; Henningsen et al., 2012; Russo et al., 2012).

These phenotype-specific dysregulated proteins independently analyzed by PRM were mainly involved in signaling, metabolic, oxidative phosphorylation, endocytosis, and proteasome pathways. Gys1 and Plcb3 were involved in the glucagon signaling pathway, Pdpk1 and Araf involved in the insulin signaling pathway, and Pak3 involved in the ErbB signaling pathway. Of note, perturbations of the glucagon and insulin signaling pathways could significantly influence the corticotropin-releasing hormone promoter gene activity in the hypothalamus, thereby adjusting the CMS-activated HPA axis (Detka et al., 2019). Atp6v1f, Atp5l, Ndufa6, Ndufb6, and Ndufv3 were involved in oxidative phosphorylation, and Pgp, Chka, Kyat1, Aldoa, Tpi1, Wasf1, Isyna1, Ctps1, and Cmpk2 were involved in several metabolic pathways. The abnormal activity of the oxidative phosphorylation system along with the diverse metabolic processes in the hypothalamus would result in an energy imbalance of the HPA axis (Nieuwenhuizen and Rutters, 2008; Harris, 2015). Furthermore, Arpc5, Arf5, and Psd were involved in endocytosis, and Psmc6, Psma1, and Psmd8 were involved in the proteasome pathway, potentially leading to aberrant hypothalamic protein trafficking and degradation. Collectively, these significant pathways have been previously implicated in the HPA axis dysfunction of stress-related mood disorders (Pittenger and Duman, 2008; Leuner and Shors, 2013; Harris, 2015). In the current research, the protein expression disturbances distinctly related to the three different behavioral phenotypes could provide a more nuanced basis for future study. Despite the determination of an imbalance of protein expressions in the hypothalamus of the stressed rats, the detailed mechanisms behind these aberrations need further investigation.

## Conclusion

In summary, we used iTRAQ- and PRM-based quantitative proteomics to analyze the CMS effect on the rat hypothalamic proteome. Our unbiased profile identified several hypothalamic protein candidates, which might be related to resiliency and vulnerability of stress-induced depression or anxiety, thus providing insights into the protein dysregulation mechanism behind CMS. The current results can serve as a significant molecular basis and deepen our understanding of the differences and similarities of stress resilience and stress-induced depression/anxiety concerning HPA axis dysfunction.

## Data Availability Statement

The proteomics raw data can be found in the ProteomeXchange Consortium with the identifier PXD022715.

## Ethics Statement

The animal study was reviewed and approved by the Ethics Committee of Chongqing Medical University.

## Author Contributions

LW and JZ designed the project and prepared the manuscript. WG, WL, CF, and YL performed the experiments. WG, CF, HX, FY, and RH analyzed the data. All authors read and approved the final manuscript.

## Conflict of Interest

CF and LW were employed by the Shenzhen Wininnovate Bio-Tech Co., Ltd. The remaining authors declare that the research was conducted in the absence of any commercial or financial relationships that could be construed as a potential conflict of interest.

## Footnotes

1 http://proteomecentral.proteomexchange.org

2 http://www.omicsbean.cn/

3 http://www.geneontology.org/

4 http://www.genome.jp/kegg/

## Supplementary Material

Supplementary Figure 1 Comparisons of the proteomic profiles of the hippocampus and hypothalamus. **(A)** Venn diagram showing the number of the total proteins quantified in each brain region. **(B–D)** The diagrams displaying the number of differential proteins in the depression-susceptible (Dep-Sus, **B**), anxiety-susceptible (Anx-Sus, **C**) and insusceptible (Insus, **D**). Hip, hippocampus; Hyp, hypothalamus.

Supplementary Figure 2 Comparison of the data from the depression-susceptible (Dep-Sus), anxiety-susceptible (Anx-Sus), and insusceptible (Insus) groups relative to the control (Cont) group between isobaric tags for relative and absolute quantitation (iTRAQ)-based and parallel reaction monitoring (PRM)-based methods.

Supplementary Figure 3 PRM analysis of the differentially regulated proteins in the depression-susceptible (Dep-Sus), anxiety-susceptible (Anx-Sus), and insusceptible (Insus) groups when compared to the control (Cont) group. The expression levels of Uqcrb, Echs1, Hspa8, Rab5a, Ephb1, Camk2a, Camk2d, Nefl, Pak1, Cbr1, Dpysl2, Tkfc, Klc4, Fyn, Flot1, Vdac1, Klc2, Snx6, and Cttn were examined on the rat hypothalamic protein extracts and shown to be altered in two or three stressed groups. *n* = 5, ^∗^*p* < 0.05, ^∗∗^*p* < 0.01.

Supplementary Table 1 Identification of the hypothalamic proteins of the depression-susceptible (Dep-Sus), anxiety-susceptible (Anx-Sus), and insusceptible (Insus) groups through the isobaric tag for relative and absolute quantitation (iTRAQ)-based proteomics analysis. The differentially regulated proteins were indicated in blue.

Supplementary Table 2 The Uniprot accessions of the proteins that comprise each region of the venn diagram in Figure 2A.

Supplementary Table 3 Enrichment results of gene ontology (GO) biological process (GOBP), cellular component (GOCC), molecular function (GOMF), and Kyoto Encyclopedia of Genes and Genomes (KEGG) pathway terms of the differentially regulated proteins from the depression-susceptible (Dep-Sus), anxiety-susceptible (Anx-Sus), and insusceptible (Insus) groups. The significantly enriched terms were highlighted in blue.

Supplementary Table 4 Enrichment results of gene ontology (GO) biological process (GOBP), cellular component (GOCC), molecular function (GOMF), and Kyoto Encyclopedia of Genes and Genomes (KEGG) pathway terms of 36 proteins that were commonly affected in all three groups. The significantly enriched terms were highlighted in blue.

Supplementary Table 5 Enrichment results of gene ontology (GO) biological process (GOBP), cellular component (GOCC), molecular function (GOMF), and Kyoto Encyclopedia of Genes and Genomes (KEGG) pathway terms of 56 proteins affected in both of the susceptible groups. The significantly enriched terms were highlighted in blue.
