# Supplementary figures and images for: Analysis of Chronic Mild Stress-Induced Hypothalamic Proteome: Identification of Protein Dysregulations Associated With Vulnerability and Resiliency to Depression or Anxiety
Source: Front Mol Neurosci. 2021 Mar 2;14:633398. doi: 10.3389/fnmol.2021.633398 (PMC7960925; doi:10.3389/fnmol.2021.633398)

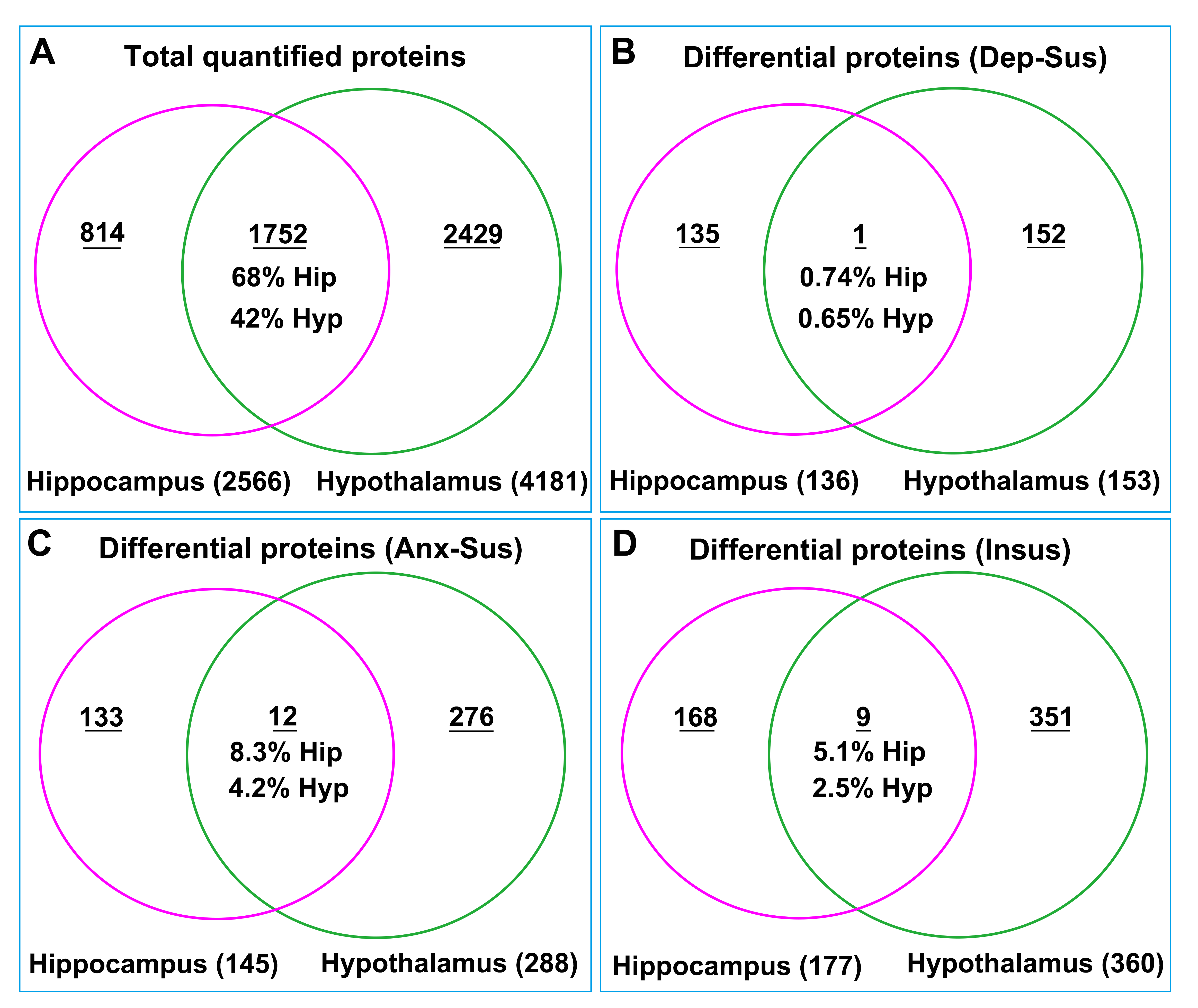

Supplement: Supplementary Figure 1 — Comparisons of the proteomic profiles of the hippocampus and hypothalamus. (A) Venn diagram showing the number of the total proteins quantified in each brain region. (B–D) The diagrams displaying the number of differential proteins in the depression-susceptible (Dep-Sus, B), anxiety-susceptible (Anx-Sus, C) and insusceptible (Insus, D). Hip, hippocampus; Hyp, hypothalamus. [file Image_1.TIF]

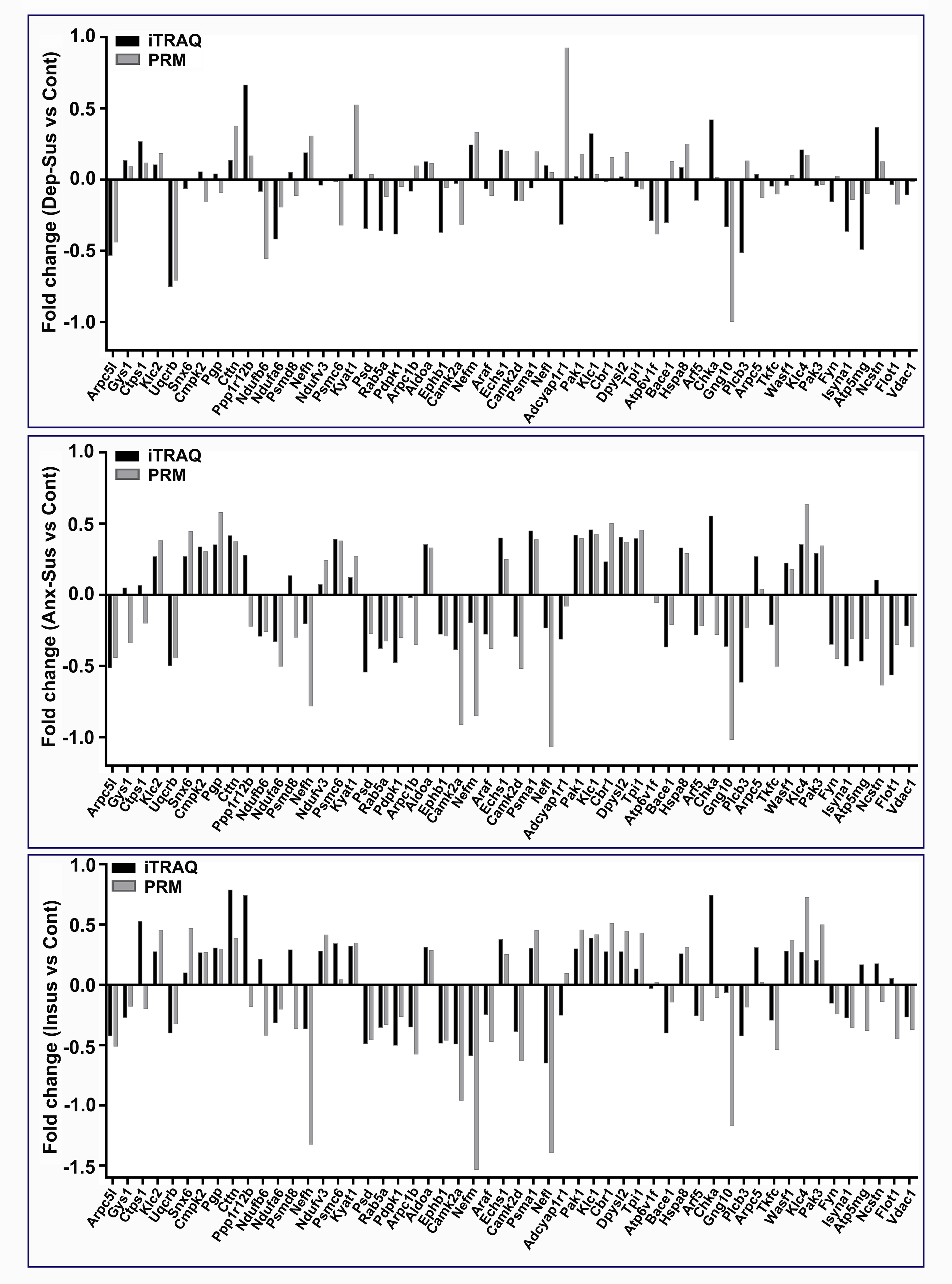

Supplement: Supplementary Figure 2 — Comparison of the data from the depression-susceptible (Dep-Sus), anxiety-susceptible (Anx-Sus), and insusceptible (Insus) groups relative to the control (Cont) group between isobaric tags for relative and absolute quantitation (iTRAQ)-based and parallel reaction monitoring (PRM)-based methods. [file Image_2.TIF]

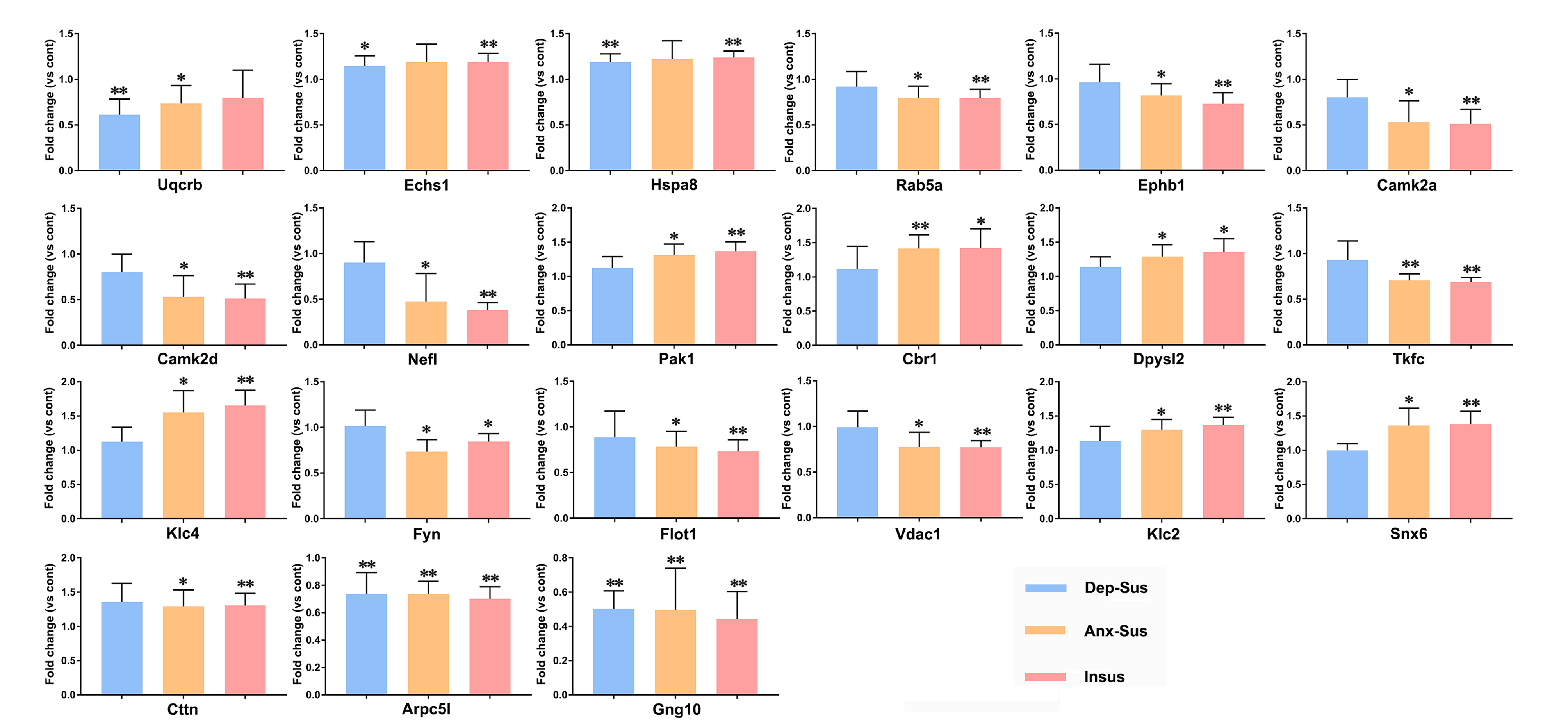

Supplement: Supplementary Figure 3 — PRM analysis of the differentially regulated proteins in the depression-susceptible (Dep-Sus), anxiety-susceptible (Anx-Sus), and insusceptible (Insus) groups when compared to the control (Cont) group. The expression levels of Uqcrb, Echs1, Hspa8, Rab5a, Ephb1, Camk2a, Camk2d, Nefl, Pak1, Cbr1, Dpysl2, Tkfc, Klc4, Fyn, Flot1, Vdac1, Klc2, Snx6, and Cttn were examined on the rat hypothalamic protein extracts and shown to be altered in two or three stressed groups. n = 5, ∗p < 0.05, ∗∗p < 0.01. [file Image_3.TIF]
